# 伴有NUP98重排的儿童急性髓系白血病9例临床分析

**DOI:** 10.3760/cma.j.cn121090-20260319-00142

**Published:** 2026-08

**Authors:** 春美 王, 晓佩 贾, 静 罗, 艳莎 张, 学聚 徐, 白 李, 叨 王, 强华 姚, 光耀 盛

**Affiliations:** 郑州大学第一附属医院儿童医院儿童血液肿瘤科，郑州 450052 Department of Pediatric Hematology and Oncology, Children's Hospital of First Affiliated Hospital of Zhengzhou University, Zhengzhou 450052, China

## Abstract

回顾性分析2019年1月至2025年6月郑州大学第一附属医院收治的9例伴有NUP98重排的急性髓系白血病（AML）患儿的临床资料。9例患儿（男3例，女6例）占同期确诊214例儿童AML的4.2％，中位诊断年龄7岁（8个月～14岁）。可见于M_1_、M_4_、M_5_亚型，以M_5_型最常见（7/9，77.8％）。融合基因类型以NUP98::NSD1最常见（6/9，66.7％），常合并FLT3-ITD、NRAS等基因突变。6例染色体核型正常，3例染色体核型异常。1个疗程诱导化疗后7例完全缓解，其中4例巩固治疗2～3个疗程后桥接异基因造血干细胞移植，均无病存活，余3例继续化疗者均复发后死亡；未完全缓解的2例因疾病进展死亡。本中心伴有NUP98重排儿童AML以女孩多见，M_5_亚型为主，NUP98::NSD1是最常见的融合基因，易合并FLT3-ITD等基因突变；诱导缓解率高但易复发，异基因造血干细胞移植可能改善其预后。

## Abstract (en)

A retrospective case analysis was conducted on nine newly diagnosed pediatric patients with NUP98-rearranged acute myeloid leukemia（AML）who were admitted to the First Affiliated Hospital of Zhengzhou University between January 2019 to June 2025. Nine patients（3 males and 6 females）accounted for 4.2% of the 214 children diagnosed with AML during the same period. The patients' median age at diagnosis was 7 years（range: 8 months to 14 years）. This type of AML was classified as M_1_, M_4_, and M_5_, with M_5_ being the most common subtype（7/9, 77.8％）. The most common fusion gene type was NUP98::NSD1（6/9, 66.7％）, which was frequently accompanied by gene mutations such as FLT3-ITD and NRAS. Six cases had normal chromosome karyotypes, whereas the remaining 3 cases had abnormal karyotypes. The complete remission（CR）rate after one course of induction chemotherapy was 77.8％（7/9）, and four of the children with CR underwent bridging allogeneic hematopoietic stem cell transplantation（allo-HSCT）after two to three courses of consolidation therapy, and all survived without disease. The remaining three children who continued chemotherapy had a relapse and died; whereas the two children who did not achieve CR died due to disease progression. In conclusion, at this center, pediatric AML with NUP98 rearrangement was more common in females and was often seen in the M_5_ subtype. NUP98::NSD1 was the most common fusion gene and was often accompanied by gene mutations, such as FLT3-ITD. Although the induction remission rate was high, the disease was prone to relaps. Allo-HSCT may improve the prognosis of these children.

急性髓系白血病（AML）是一类起源于造血干祖细胞的恶性克隆性疾病，占儿童急性白血病的15％～20％[1]。近年来，随着风险适应性治疗方案的不断优化，其总生存（OS）率约70％[2]–[3]，但不同分子亚型的AML仍有较大差异。核孔蛋白98（nuclearporin98, NUP98）基因位于染色体11p15，由33个外显子组成，编码的NUP98蛋白属核孔复合物的一部分，对细胞有丝分裂、核浆转运等有调控作用[4]。NUP98重排可通过干扰造血细胞的转录调控促进AML发生。目前报道的NUP98重排可与30多种伴侣基因形成融合基因[5]，已被列入儿童AML的高危因素之一。儿童AML中NUP98重排相对罕见，约占5％[6]，与儿童AML的高恶性程度及治疗反应差相关。目前有关国内儿童NUP98重排AML的临床报道较少，现报道我中心2019年1月至2025年6月收治的9例伴有NUP98重排的AML儿童病例，总结其临床特点、治疗及预后情况，以期为该类患儿的临床诊治提供临床经验。

## 病例与方法

1. 病例资料：回顾性分析2019年1月至2025年6月郑州大学第一附属医院儿童血液肿瘤内科收治的124例AML患儿的临床资料。所有患者均结合骨髓细胞形态学、免疫学、细胞遗传学、分子生物学（MICM）进行诊断分型，诊断及危险度分型均符合《儿童急性髓系白血病诊疗专家共识（2024）》[7]。本研究获得郑州大学第一附属医院伦理委员会审核批准（批件号：2026-KY-0352-001）。

2. 治疗方案：根据患儿病情及AML方案的更新情况选择合适的初次诱导缓解方案，DA、DAE方案各1例，DAH、VAH方案各2例，MAG方案3例。具体用药如下：DA方案：柔红霉素（DNR）40 mg·m^−2^·d^−1^，第1～3天；阿糖胞苷（Ara-C）200 mg·m^−2^·d^−1^，第1～7天，分2次给予。DAE方案：DNR 50 mg·m^−2^·d^−1^，第2、4、6天；Ara-C 100 mg·m^−2^·次^−1^，每12 h 1次，第1～7天；依托泊苷（Vp16）100 mg·m^−2^·d^−1^，第1～5天。DAH方案：DNR 40 mg·m^−2^·d^−1^，第1、3、5天；Ara-C 100 mg·m^−2^·次^−1^，每12 h 1次，第1～7天；高三尖杉酯碱（HHT）3 mg·m^−2^·d^−1^，第1～5天。VAH方案：HHT 3 mg·m^−2^·d^−1^，第1～5天；Ara-C 100 mg·m^−2^·次^−1^，每12 h 1次，第1～7天；维奈克拉50 mg·m^−2^·d^−1^，第1天；100 mg·m^−2^·d^−1^，第2天；200 mg·m^−2^·d^−1^，第3～28天。MAG方案：米托蒽醌（Mit）5 mg·m^−2^·d^−1^，第1、3、5天；Ara-C 10 mg·m^−2^·次^−1^，每12 h 1次，第1～10天；G-CSF 5 µg·kg^−1^·d^−1^，第1～10天（若外周血WBC>5.0×10^9^/L，可暂不应用G-CSF）。

3. 疗效评估及随访：于每轮诱导治疗第28天、巩固治疗后第28天、维持治疗期间每3个月及停药时评估骨髓缓解状态、可检测残留病（MRD）水平。骨髓完全缓解（CR）定义为骨髓原始细胞比例<5％。复发定义为CR后外周血再次出现白血病细胞或骨髓中原始细胞≥5％（除外巩固化疗后骨髓再生等其他原因），或髓外出现白血病细胞浸润。骨髓MRD转阴指MRD<0.01％。OS期指自诊断日至死亡或末次随访的时间。对所有患儿通过电话或查阅病例资料进行随访，随访截止日期为2025年12月31日。

4. 统计学处理：采用描述性分析，非正态分布的计量资料采用中位数（范围）表示；计数资料采用例数和百分率（％）表示。

## 结果

1. 临床特征：同期就诊于我院的年龄≤14岁的儿童AML患者共214例，伴有NUP98重排AML患儿9例，占4.2％，其中男3例，女6例，中位年龄7岁（8个月～14岁）。按FAB分型主要为M_5_型（7/9，77.8％），M_1_型和M_4_型各1例。初诊时6例患儿以发热起病，4例患儿合并淋巴结肿大，2例患儿伴有面部肿物，1例患儿伴有肝脏肿大。4例患儿初诊时存在高白细胞血症（WBC>100×10^9^/L），患儿临床特征详见表1。

**表1:** 9例伴有NUP98重排的急性髓系白血病患儿的基本情况与临床特征

例号	年龄（岁）	性别	WBC（×10^9^/L）	FAB分型	NUP98重排类型	染色体核型	基因突变	治疗方案	诱导治疗疗效	诱导后MRD	是否移植（移植前巩固疗程）	转归
1	7	女	294.67	M_1_	NUP98::NSD1	46，XX[20]	ASXL1、KRAS、FLT3-ITD	DAH	NR	阳性	否	死亡
2	1	男	12.81	M_5_	NUP98::PHF23	46，XY,+1,psu dic（1:9）（q34:p13）[15]/46,XY[5]	NRAS	DA	CR	未测	否	死亡
3	12	女	315.70	M_5_	NUP98::NSD1	46，XX[20]	FLT3-ITD	DAH+索拉非尼	CR	阴性	是（3个）	存活
4	13	男	73.35	M_5_	NUP98::NSD1	46，XY[20]	WT1、FLT3-ITD	DAE+索拉非尼	CR	阴性	否	死亡
5	1	女	50.30	M_4_	NUP98::KDM5A	46，XX[20]	NRAS	MAG	PR	阳性	否	死亡
6	8个月	男	5.07	M_5_	NUP98::KDM5A	46，XY，add（9）（p24）;del（q12;q14）[11]/46,XY[17]	无	MAG	CR	阴性	是（2个）	存活
7	4	女	211.90	M_5_	NUP98::NSD1	48,XX,+8,+9[11]	FLT3-ITD	MAG+索拉非尼	CR	阴性	否	死亡
8	9	女	63.38	M_5_	NUP98::NSD1	46，XX[20]	NRAS、WT1、FLT3-ITD、RUNX1	VAH+吉瑞替尼	CR	阴性	是（3个）	存活
9	11	女	107.75	M_5_	NUP98::NSD1	46，XX[2]	WT1、FLT3-ITD	VAH+吉瑞替尼	CR	阳性	是（2个）	存活

**注** DA：柔红霉素（DNR）+阿糖胞苷（Ara-C）；DAE：DNR +Ara-C +依托泊苷；DAH：DNR+Ara-C+高三尖杉酯碱；VAH：HHT+Ara-C +维奈克拉；MAG：米托蒽醌 +Ara-C+G-CSF；NR：未缓解；CR：完全缓解；PR：部分缓解；MRD：可检测残留病

2. 分子生物学及细胞遗传学特征：9例伴有NUP98重排AML患儿中，6例NUP98::NSD1阳性，2例NUP98::KDM5A阳性，1例NUP98::PHF23阳性。二代测序（NGS）结果显示，8例NUP98重排AML患儿同时合并基因突变，其中6例NUP98::NSD1阳性患儿均合并FLT3-ITD突变，同时还可见WT1、ASXL1、KRAS、NRAS、RUNX1突变；2例NUP98::KDM5A阳性的患儿中1例合并NRAS突变；1例NUP98::PHF23阳性患儿合并NRAS突变（表1）。

染色体核型分析结果显示，6例染色体核型正常，其中5例为NUP98::NSD1阳性患儿，1例为NUP98::KDM5A阳性患儿；3例染色体异常；NUP98::NSD1阳性、NUP98::KDM5A阳性及NUP98::PHF23阳性患儿各1例（表1）。

3. 治疗及预后：9例患儿中，7例1个疗程诱导治疗后达CR，其中DA、DAE、DAH方案各1例，MAG、VAH方案各2例，其中5例同时联合索拉非尼或者吉瑞替尼靶向治疗；5例MRD阴性，1例MRD阳性，1例未测。CR的患儿中4例经巩固治疗2～3个疗程后桥接异基因造血干细胞移植（allo-HSCT），其中3例于移植前骨髓形态学CR，MRD及融合基因均阴性，1例（例8）移植前达骨髓形态学CR，但MRD及融合基因阳性，截至随访日期，均无病存活，余3例继续化疗的患儿分别于治疗后9个月余、10个月余、1年余复发死亡；1例部分缓解的患儿于诊断后6个月余因疾病进展死亡，1例未缓解的患儿于诊断后3个月余因疾病进展死亡，中位OS期为10.0（3.5～55.7）个月（表1）。

## 讨论

细胞遗传学和分子生物学异常在儿童AML的危险分层中起重要作用，是影响预后的重要因素。NUP98重排的儿童AML是一种特殊亚型，由于该类患儿缓解率和OS率低，目前纳入儿童AML的高危因素之一。NUP98重排在儿童AML中的发生率低，约为5％[6],[8]，也有文献报道为10％左右[9]。该类患儿发病年龄偏大，中位发病年龄为10.3岁，男孩多见[6]，初诊时常伴有WBC增高，FAB分型以M_4_/M_5_亚型多见，常常合并FLT3-ITD突变，尤其是NUP98::NSD1阳性的AML患儿居多。本中心NUP98重排AML患儿的发生率为4.2％，发病中位年龄为9岁，7例患儿WBC>50×10^9^/L，其中4例患儿WBC>100×10^9^/L，7例为M_5_，以上结果和既往报道相符。但我中心患儿以女孩多见，考虑与单中心数据存在数据偏倚有关，后续将进一步扩大样本量进行研究。

NUP98可以与多种伙伴基因形成融合基因，目前已有超过30种NUP98融合伴侣被发现，如NUP98::HOX、NUP98::NSD1、NUP98::KDM5A、NUP98::PHF23等，可见于多种血液系统恶性肿瘤中，包括AML、骨髓增生异常肿瘤、急性T淋巴细胞白血病、混合细胞型白血病等，其中以儿童AML最为常见[5]。不同于成人AML中NUP98重排的类型主要为NUP98::HOX，儿童NUP98重排的类型主要为NUP98::NSD1。Struski等[10]的研究显示，574例儿童AML患者中22例检测到NUP98重排，最常见的NUP98融合基因伴侣是NSD1。Chisholm等[11]分析24例儿童急性红系白血病患者，其中7例NUP98阳性，最常见的NUP98融合基因伴侣是NSD1。Swaminathan等[12]的研究显示，16例NUP98重排AML患者中，14例为成人，2例为儿童，所有的融合基因伴侣均为NSD1。本研究中9例儿童NUP98重排患者的类型主要为NUP98::NSD1，占66.7％，和既往研究相符。

伴NUP98重排的AML患者似乎有独特的基因突变谱。Pigazzi等[13]的一项来自AIEOP AML-2002的研究表明，50％的NUP98::NSD1阳性AML患者伴有FLT3-ITD突变。Hollink等[14]的研究表明，23例NUP98::NSD1阳性的AML患者中，FLT3-ITD突变者占91％，WT1突变者占45％。Bertrums等[6]的研究表明，NUP98::NSD1和NUP98::KDM5A阳性的AML患者中FLT3-ITD突变者占74％，WT1突变者占42％。亦有研究表明，部分NUP98重排的AML患者合并KRAS、NRAS、ASXL1、KIT、MYC、RUNX1突变[15]–[16]，本研究发现，6例NUP98::NSD1阳性患儿均合并FLT3-ITD突变，3例同时合并WT1突变，部分患儿同时合并ASXL1、KRAS、NRAS、RUNX1突变，和既往研究相符。复杂核型在NUP98重排的AML患者中较为少见，且许多患者如NUP98::NSD1阳性患者常表现为正常的染色体核型，但在一些特殊亚型的AML中，如NUP98::KDM5A阳性的AML-M_7_患儿中，常伴有染色体异常[17]。本研究发现，9例AML患儿中，6例染色体核型正常，其中5例为NUP98::NSD1阳性的患儿，1例为NUP98::KDM5A阳性的患儿；3例染色体异常，包括NUP98::NSD1阳性、NUP98::KDM5A阳性及NUP98::PHF23阳性患儿各1例，均为非复杂核型，与既往研究类似。

既往研究提示，伴有NUP98重排的AML患者的诱导缓解率低，复发率高。Struski等[10]研究发现，22例伴有NUP98重排的儿童AML中，诱导治疗后CR率和治疗后5年OS率分别为48％和67％，明显低于对照组（NUP98基因重排阴性者）的89％和72％。王彤等[18]的研究发现，8例NUP98::NSD1阳性的儿童AML中，3例患者经多个疗程化疗始终不缓解，5例患者获得血液学缓解但于1～19个月后复发，5例患者进行了挽救性allo-HSCT，1例存活。Niktoreh等[2]报道15例合并WT1、FLT3-ITD突变的NUP98::NSD1阳性的儿童AML患者，诱导缓解率低，OS率仅27％。Huang等[15]的研究显示，128例NUP98融合基因阳性的患者（包括87例成人）中，NUP98::NSD1组的CR率显著低于非NSD1组（50.8％对72.5％），其中42例患者接受了allo-HSCT，除了两例复发病例外，所有患者均实现了持续的MRD阴性。1例NUP98::NSD1阳性患者在化疗失败后接受menin抑制剂后实现了MRD阴性和长期无病生存。Chen等[19]采用Palbociclib联合维奈克拉和阿扎胞苷治疗NUP98重排的儿童AML，9例患儿中6例达CR，且MRD阴性，所有具有NUP98::NSD1融合基因的患者最终都表现出完全分子学缓解，而具有NUP98::KDM5A或NUP98::TOP1融合基因的患者则分子学持续阳性，2年OS和EFS率分别为88.9％和77.8％。本研究中9例患儿经过1个疗程诱导治疗后7例达CR，高于既往传统化疗组，其中DA、MAG方案各1例，DAH+索拉非尼1例，DAE+索拉非尼1例，MAG+索拉非尼1例，VAH+吉瑞替尼2例，提示目前中国儿童常用的AML方案联合FLT3抑制剂均适用于NUP98重排的患者，可根据患儿的疾病状态选择合适的治疗方案。CR的患儿中4例经巩固治疗2～3个疗程后桥接allo-HSCT，均无病存活，余3例继续化疗及2例首次诱导治疗后未达CR的患儿均因疾病复发或者进展死亡，提示对于该类患儿，首次CR后尽早桥接allo-HSCT可改善预后。

综上，本中心NUP98重排在儿童AML中发生率为4.2％，女孩多见，常见于M_5_亚型，以NUP98::NSD1最常见，易合并FLT3-ITD等基因突变；虽诱导缓解率高，但易复发，allo-HSCT可能改善该类患儿的预后。然而，本研究是单中心回顾性病例研究，病例数较少，可能存在数据偏倚，后续需进行多中心、大样本的研究进一步探讨。
